# Endosomal protein expression of γ1-adaptin is associated with tumor growth activity and relapse-free survival in breast cancer

**DOI:** 10.1007/s12282-023-01539-1

**Published:** 2024-01-24

**Authors:** Nobuhiro Hoshi, Takefumi Uemura, Kazunoshin Tachibana, Sadahiko Abe, Yuko Murakami-Nishimagi, Maiko Okano, Masaru Noda, Katsuharu Saito, Koji Kono, Tohru Ohtake, Satoshi Waguri

**Affiliations:** 1 Department of Breast Surgery, Fukushima Medical University School of Medicine, Fukushima, Japan; 2 Department of Anatomy and Histology, Fukushima Medical University School of Medicine, Fukushima, Japan; 3 Department of Gastrointestinal Tract Surgery, Fukushima Medical University School of Medicine, Fukushima, Japan

**Keywords:** γ1-Adaptin, Clathrin adaptor, Adaptor protein complex-1, AP-1, Endosome

## Abstract

**Background:**

γ1-Adaptin is a subunit of adaptor protein complex-1 (AP-1), which regulates intracellular transport between the trans-Golgi network (TGN) and endosomes. Since expression levels of AP-1 subunits have been reported to be associated with cell proliferation and cancer malignancy, we investigated the relationships between the immunohistochemical expression of γ1-adaptin and both clinicopathological factors and relapse-free survival (RFS) in breast cancer tissue.

**Materials and methods:**

SK-BR-3 cell line depleted of γ1-adaptin was used for cell proliferation, migration, and invasion assay. Intracellular localization of γ1-adaptin was examined with immunohistochemistry (IHC) using an antibody against γ1-adaptin, and with double immunohistofluorescence (IHF) microscopy using markers for the TGN and endosome. γ1-Adaptin intensities in IHC samples from 199 primary breast cancer patients were quantified and assessed in relation to clinicopathological factors and RFS.

**Results:**

Cell growth, migration, and invasion of SK-BR-3 cells were significantly suppressed by the depletion of γ1-adaptin. Although the staining patterns in the cancer tissues varied among cases by IHC, double IHF demonstrated that γ1-adaptin was mainly localized in EEA1-positive endosomes, but not in the TGN. γ1-Adaptin intensity was significantly higher in the tumor regions than in non-tumor regions. It was also higher in patients with Ki-67 (high), ER (–), PgR (–), and HER2 (+). Among subtypes of breast cancer, γ1-adaptin intensity was higher in HER2 than in luminal A or luminal B. The results of the survival analysis indicated that high γ1-adaptin intensity was significantly associated with worse RFS, and this association was also observed in group with ER (+), PgR (+), HER2 (–), Ki-67 (high), or luminal B. In addition, the Cox proportional hazards model showed that high γ1-adaptin intensity was an independent prognostic factor.

**Conclusion:**

These results suggest that the endosomal expression of γ1-adaptin is positively correlated with breast cancer malignancy and could be a novel prognostic marker.

## Introduction

Breast cancer is the leading cause of cancer death among females worldwide, according to GLOBOCAN 2020 estimates [1]. It is a complex disease, and currently classified into four subtypes: luminal A, luminal B, human epidermal growth factor receptor type 2 (HER2)-positive, and triple-negative breast cancer (TNBC). They reflect intrinsic biological subtypes [2] determined by the immunohistochemistry (IHC) of estrogen receptor (ER), progesterone receptor (PgR), HER2, and a cell proliferation marker Ki-67 [3, 4]. Importantly, these subtypes are strongly associated with treatment strategies and prognosis; e.g., TNBC shows poorer prognosis than the luminal types of breast cancer [5, 6]. However, late recurrence was higher in patients with ER-positive primary tumors [7] and pathological complete response following preoperative chemotherapy in TNBC patients was associated with good prognosis [8, 9]. Therefore, the development of more effective diagnostic and/or prognostic markers is required to improve appropriate personalized medicine for breast cancer patients.

Adaptor protein complex-1 (AP-1) is one of clathrin adaptor molecules regulating intracellular transport of specific cargos between the trans-Golgi network (TGN) and endosomes. It is a heterotetramer complex in human consisting of β1-, γ-, μ1- and σ1-adaptins, with two isoforms of γ-adaptin (γ1 and γ2) and μ1-adaptin (μ1A and μ1B), and three isoforms of σ1-adaptin (σ1A, σ1B, and σ1C). The head domain of γ-adaptin interacts with active ADP ribosylation factor 1 (ARF1), which is responsible for the recruitment of AP-1 on the organelle membrane. Hinge regions of β1- and γ-adaptins bind clathrin, while μ1-adaptin interacts with cargo proteins, contributing to their efficient sorting. The best-known cargo is the mannose 6-phosphate receptors (MPRs) that transport newly synthesized lysosomal enzymes from the TGN into the endo-lysosomal system. In addition, AP-1 is reportedly involved in the endosomal transport of transferrin receptor, low-density lipoprotein receptor, and epidermal growth factor receptor (EGFR) [10–13]. It was previously reported that the upregulation of AP-1 mediated by Hepatitis B virus-transfection promotes cell proliferation of HepG2, a cell line derived from hepatocellular carcinoma [14], and that μ1-adaptin could be one of prognostic markers for central nervous system metastasis of TNBC [15]. In addition, studies focusing on gene expression of *AP1S3* that encodes σ1C revealed that it is highly expressed in TNBC, glioma, and pancreatic ductal adenocarcinoma (PDAC) [16–18], suggesting high expression of AP-1 complex in these cancers. We have recently reported that AP-1 containing γ1-adaptin could support cancer growth by maintaining cell surface expression of EGFR, and that γ1-adaptin-positive granular staining is often observed in tissues of hepatocellular carcinoma, non-small-cell lung carcinoma (NSCLC), and colorectal adenocarcinoma [13]. Thus, protein expression of γ1-adaptin could also support the growth of breast cancer. In the present study, to investigate whether γ1-adaptin is associated with breast cancer malignancy, we examined its expression using immunohistological analyses, as well as the associations of its intensity with both clinicopathological factors and relapse-free survival (RFS).

## Materials and methods

### Human tissue samples

Tumors were surgically resected from patients with breast cancer at Fukushima Medical University Hospital (Fukushima, Japan). Cases with de novo Stage IV and those treated with preoperative chemotherapy were excluded from this study. Paraffin-embedded tissue samples of 199 cases were obtained between 2011 and 2014, which were used for IHC, while both paraffin-embedded and frozen tissue samples from 14 patients were obtained between April 2020 and July 2020. Regarding the frozen samples, a portion of each tissue specimen was snap-frozen immediately after resection and stored at -80˚C until use. This study was approved by the Ethics Committee of Fukushima Medical University (Number 3181). Informed consent was obtained from all individual participants included in the study. Breast cancer subtypes were determined based on the expression of ER, PgR, and HER2, and Ki-67, with a cut-off point of 14% [19].

### Cells and RNAi experiment

SK-BR-3, MCF-7, and HEK293T cells were purchased from ATCC. They were grown in Dulbecco’s modified Eagle’s medium (DMEM; Nacalai Tesque, Kyoto, Japan) supplemented with 10% fetal bovine serum (FBS) at 37 °C in 5% CO_2_.

For constitutive knockdown of γ1-adaptin, the pLKO.1 puro vector (Sigma) containing γ1-adaptin shRNA (5ʹ-AGGAAGUUAUGUUCGUGAU-3ʹ) was constructed according to the manufacturer's instructions. Subsequently, HEK293T cells were transfected with γ1-adaptin shRNA vector or pLKO.1 empty vector as a control for 48 h, and virus-containing media were then collected. SK-BR-3 cells were infected with the lentivirus in DMEM containing 10% FBS and 8 μg/ml polybrene for 48 h, and media were replaced with fresh media containing puromycin at concentrations of 3 μg/ml. Following confirmation of knockdown efficiency in Western blotting experiments, the bulk of puromycin-resistant cells was used for in vitro cell growth, migration, and invasion assays.

For a preparation of paraffin-embedded cell pellets, MCF-7 cells were transfected with siRNAs for γ1-adaptin (5ʹ-AGGAAGUUAUGUUCGUGAU-3ʹ) or control siRNA (Silencer Negative Control #1 siRNA [AM4611, Ambion, TX, USA]) using Lipofectamine RNAiMAX Transfection Reagent (Thermo Fisher Scientific, MA, USA) as described previously [20, 21]. Three days later, the cells were fixed with 4% paraformaldehyde and 4% sucrose in 0.1 M PBS for 30 min at room temperature. After being washed three times with 0.1 M phosphate buffer (pH 7.4), they were collected with a scraper, and then embedded in iPGell (Genostaff, Japan) for paraffin embedding.

### Western blot analysis

Western blot analysis of tissue samples was conducted as previously reported [20]. Briefly, frozen tissues of breast cancer were homogenized in PBS containing 1% Triton X-100, a protease inhibitor cocktail (Roche), and phosphatase inhibitors (Roche). Lysates were then separated on 5–20% gradient gels (Wako), and were transferred to PVDF membranes (Millipore). After blocking with PBS containing 5% skim milk and 0.1% Tween 20 for 30 min, the membranes were incubated with the primary antibodies against γ1-adaptin antibody (BD Transduction Laboratories [610385]), GAPDH (Santa Cruz Biotechnology [sc-32233]), or EGFR (Cell Signaling Technology [4267]). For signal detection, ECL Prime (GE Healthcare) and ImageQuant LAS 4000 mini (GE Healthcare) were used. The signal intensities of the bands were quantified using Image J, and the values of γ1-adaptin were normalized with those of GAPDH.

### Analyses of cell growth, migration, and invasion in vitro

For cell growth assay, cells were seeded on 96-well plates in hexaplicate at 500 cells per well. Cells were allowed to grow for 24 h, and were then cultured for 1, 4, 7, and 10 days. Cell growth assays were performed using Cell Counting Kit-8 (Dojindo) according to the manufacturer’s instructions. OD450 value was measured, and the ratio to the value at day1 was plotted as the means ± SD of three experiments. The statistical differences were analyzed using Student’s *t* test. For cell migration and invasion assays, CytoSelect™ 24-Well Cell Migration and Invasion Assay (Cell Biolabs, Inc., CBA-100-C, CA, USA) was used according to the manufacture’s instruction. Briefly, cell suspensions containing 1.0 × 10^6^ cells/ml in DMEM were prepared, and 300 μl of each suspension was added to insert chambers that had been placed in 24 well plates. Three-hundred (300) μl of DMEM containing 10% FBS was added to the well with the insert chamber. After 24 h culture, cells were stained with Cell Stain Solution, washed with water, and lysed with Extraction Solution. Cell migration and invasion were quantified at OD 560 nm in a plate reader. The ratio of sh-γ1 to Ctrl was plotted as mean ± SD of three experiments. The statistical difference was analyzed using Student’s *t *test.

### Immunohistochemistry

Sections of 3 µm-thickness were prepared from paraffin-embedded tissue samples from breast cancer patients. After deparaffinization, they were processed for antigen retrieval for 20 min at 98 °C using a microwave processor (MI-77, AZUMAYA, Japan) in 1% immunosaver (Nissin EM, Japan), inactivation of endogenous peroxidase by incubation with 0.3% H_2_O_2_ in methanol for 20 min at room temperature, and blocking of nonspecific binding by incubation with 10% goat serum (Jackson ImmunoReseach Inc, PA, USA) for 20 min at room temperature. They were further incubated with anti-γ1-adaptin antibody (BD Bioscience, HJ, USA; 1:1,000 dilution) for 2 days at 4 °C, and then with peroxidase-labeled anti-mouse antibody (Histofine Simple stain MAX-PO (M), Nichirei Biosciences Inc., Japan) for 30 min at room temperature. Peroxidase activity was detected with 0.0125% 3,3ʹ-diaminobendizine and 0.002% H_2_O_2_ in 0.05 M Tris–HCl buffer (PH 7.6). After being stained with hematoxylin as nuclear counter staining, they were mounted on glass slides and observed with a microscope (BX51, Olympus, Japan) equipped with a cooled CCD camera system (DP-71, Olympus). For quantification, the nuclear counter staining was omitted, and five reference cases were included in every experiment for data correction between the experiments.

### Immunohistofluorescence (IHF) microscopy

Sections of the paraffin-embedded cell pellets and the breast cancer tissues were prepared and processed for antigen retrieval as above. They were processed for IHF as described previously [13]. After being blocked with 10% goat serum for 20 min at room temperature, they were incubated with anti-γ1-adaptin antibody (BD Bioscience; 1:1000) alone for cell pellets, or with a mixture of anti-γ1-adaptin antibody and either rabbit anti-EEA1 antibody (Abcam, Cambridge, UK; 1:200 dilution) or sheep anti-human TGN46 (Bio-Rad Laboratories, Inc., CA, USA, 1:400 dilution) for patient specimens, for 1 day at 4 °C. They were then incubated with Alexa 594-conjugated donkey anti-mouse IgG (1:800 dilution), or a mixture of Alexa 594-conjugated donkey anti-mouse IgG and Alexa 488-conjugated donkey anti-rabbit IgG for 60 min at room temperature. They were observed with a confocal laser microscope (FLUOVIEW FV1000, OLYMPUS, Japan).

### Image analysis for quantification

Images of γ1-adaptin-stained specimens by IHC were captured with a virtual slide scanner (Nanozoomer-SQ, Hamamatsu photonics, Japan) equipped with a × 40 lens, which were observed with a piece of software, NDP.view 2 (Hamamatsu photonics, Japan). Quantification was performed using Fiji software (National Institute of Health, MD, USA) according to Okabe et al. [22] with some modifications (Fig. 3a–c). Three ROIs of rectangle areas (198 × 318 µm) containing relatively strong immunoreactivities were selected per specimen. In each ROI, an area mainly containing tumor cells was manually enclosed, and then, stained pixels were extracted by thresholding the intensity. The sum of pixel intensities was then divided by the enclosed area, which was considered the γ1-adaptin intensity of the ROI. The average of the three ROIs was taken as a value of each case. Differences in γ1-adaptin intensity between experiments were corrected by including five identical cases in all experiments as references. For 44 non-cancerous mammary gland tissues, a single ROI was used for quantification.

### Statistical analysis

Pearson's Chi-squared test was used for evaluating correlations of γ1-adaptin expression with clinicopathological factors. The Mann–Whitney *U* test and Steel–Dwass test were used for comparison analyses between the two groups and more than three groups, respectively. Survival analyses were performed using the Kaplan–Meier methods with the log-rank test, and by Cox proportional hazards model. Student’s *t* test was used for the cell growth, migration, and invasion assays. GraphPad Prism7 (GraphPad Software, CA, USA) and R software were used for all statistical analyses. *p* values of < 0.05 were considered statistically significant.

## Results

### Depletion of γ1-adaptin suppresses the growth of SK-BR-3 cells

**Fig. 1:**
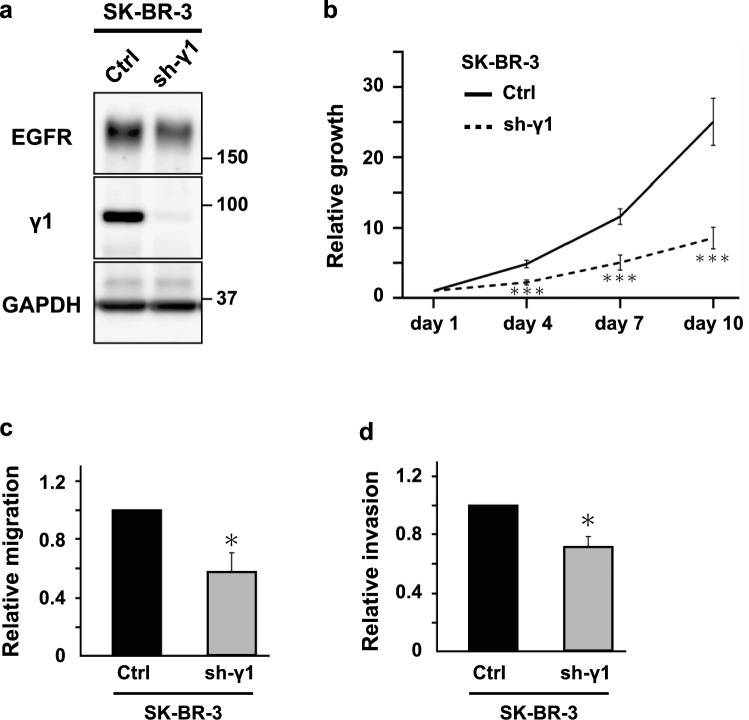
Effect of γ1-adaptin depletion on cell growth, migration, and invasion. **a** Western blot analysis of control (Ctrl) and γ1-adaptin-depleted SK-BR-3 cells (sh-γ1) using antibodies against EGFR, γ1-adaptin (γ1), and GAPDH. Molecular weight is indicated on the right. **b** Ctrl and sh-γ1 cells were cultured for 1, 4, 7, and 10 days, and their cell growth activity was assessed. The ratio to the value at day1 was plotted as the means ± SD of three experiments. The statistical differences between Ctrl and sh-γ1 at each time point were analyzed using Student’s *t* test. ***p < 0.001. **c**, **d** Cell migration (**c**) and invasion (**d**) of Ctrl and sh-γ1 cells were analyzed. The ratio of sh-γ1 to Ctrl was plotted as mean ± SD of three experiments. The statistical difference was analyzed using Student’s *t* test (*p < 0.05)

To know whether depletion of γ1-adaptin affects cancer cell properties, we examined HER2-positive breast cancer cell line, SK-BR-3. γ1-Adaptin was remarkably depleted by the constitutive knockdown of γ1-adaptin shRNA (Fig. 1a), and as reported [13], EGFR was concomitantly decreased (Fig. 1a). Growth rates, and cellular migration and invasion activities of the γ1-adaptin-depleted SK-BR-3 cells were significantly lower than those of control cells (Fig. 1b–d). These findings suggest that γ1-adaptin expression supports cancer cell activities, prompting us to investigate the expression levels of γ1-adaptin in breast cancer tissues.

### Expression and localization of γ1-adaptin in breast cancer tissues

**Fig. 2:**
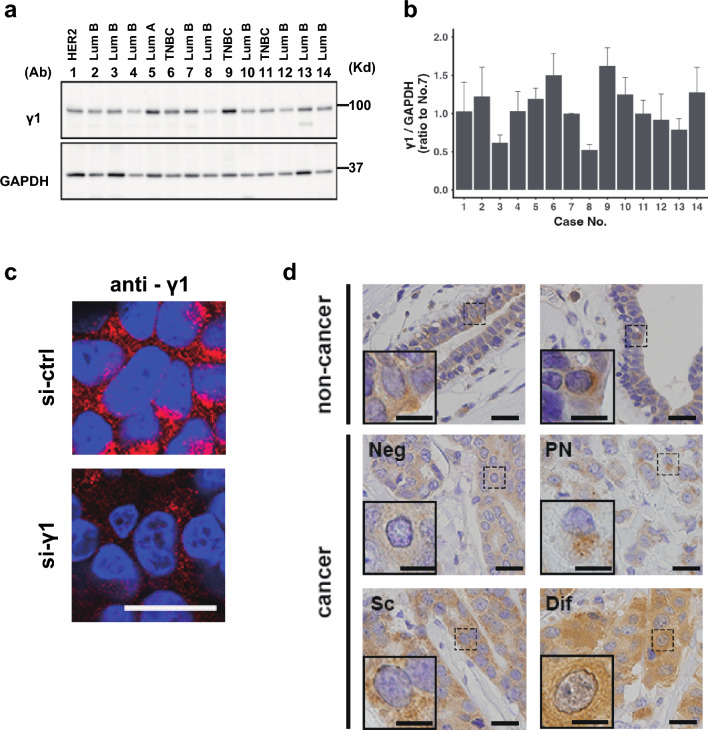
Protein expression of γ1-adaptin in breast cancer tissues. **a** Lysates of 14 cases of breast cancer were examined by Western blot analysis using anti-γ1-adaptin (γ1) and GAPDH antibodies. Subtypes are indicated on the top. Lum: Luminal. Molecular weight (Kd) is indicated on the right. **b** The band intensity of γ1 was normalized to that of GAPDH, and the ratio to the value of No. 7 is plotted as mean ± SD (from three experiments). The case numbers correspond to those in (**b**). **c** To validate anti-γ1 antibody in IHC, cell pellets of MCF-7 cells transfected with control siRNA (si-ctrl) or siRNA for γ-adaptin (si-γ1) were immunostained using anti-γ1 (anti-γ1) (red). The nuclei were stained with Hoechst 33342 (blue). Bar: 20 µm. **d** Localization of γ1-adaptin in the non-cancer and cancer regions of breast cancer tissues are shown. For the cancer regions, four staining patterns were observed: Neg (negative), PN (perinuclear), SC (scattered), and Dif (diffuse). The nuclei were stained with hematoxylin. Boxed regions are magnified and shown in insets. Bars: 20 µm

We first examined γ1-adaptin expression in 14 breast cancer tissues by Western blot analysis. As shown in Fig. 2a, b, amounts of γ1-adaptin varied among tissues. To examine γ1-adaptin distribution by IHC, the applicability of this antibody to paraffin-embedded tissue was evaluated by immunofluorescence microscopy in paraffin-embedded cell pellets of MCF-7. The antibody clearly stained perinuclear Golgi region and cytoplasmic peripheral puncta, but the signal was drastically reduced in the cells treated with siRNA for γ1-adaptin (Fig. 2c). Because a similar reduction has been observed in a lung cancer cell line, H1975 [13], we conclude that this antibody is applicable for IHC using paraffin-embedded tissues. In humans, there are γ1- and γ2-adaptins, which are encoded by the AP1G1 and AP1G2 genes, respectively. Our previous work showed that this antibody specifically recognizes γ1-adaptin and that depletion of γ1-adaptin using siRNA decreased other subunits of AP-1 complex except γ2-adaptin [13]. Thus, the signal most likely reflects AP-1 containing γ1-adaptin rather than γ2-adaptin.

By conventional IHC with DAB staining, γ1-adaptin-positive puncta were localized mainly in the supra-nuclear region, and sparsely in the other cytoplasmic regions in the ductal epithelial cells of normal mammary grands found in non-cancer regions (Fig. 2d). On the other hand, the cellular staining pattern in the cancer tissues varied among cases, and could be classified into four groups in terms of the presence and distribution of the puncta: no significant staining (Neg), perinuclear dominant puncta (PN), scattered puncta (SC), and no puncta but diffuse staining (Dif) (Fig. 2d).

### Endosome-dominant localization of γ1-adaptin in breast cancer cells

**Fig. 3:**
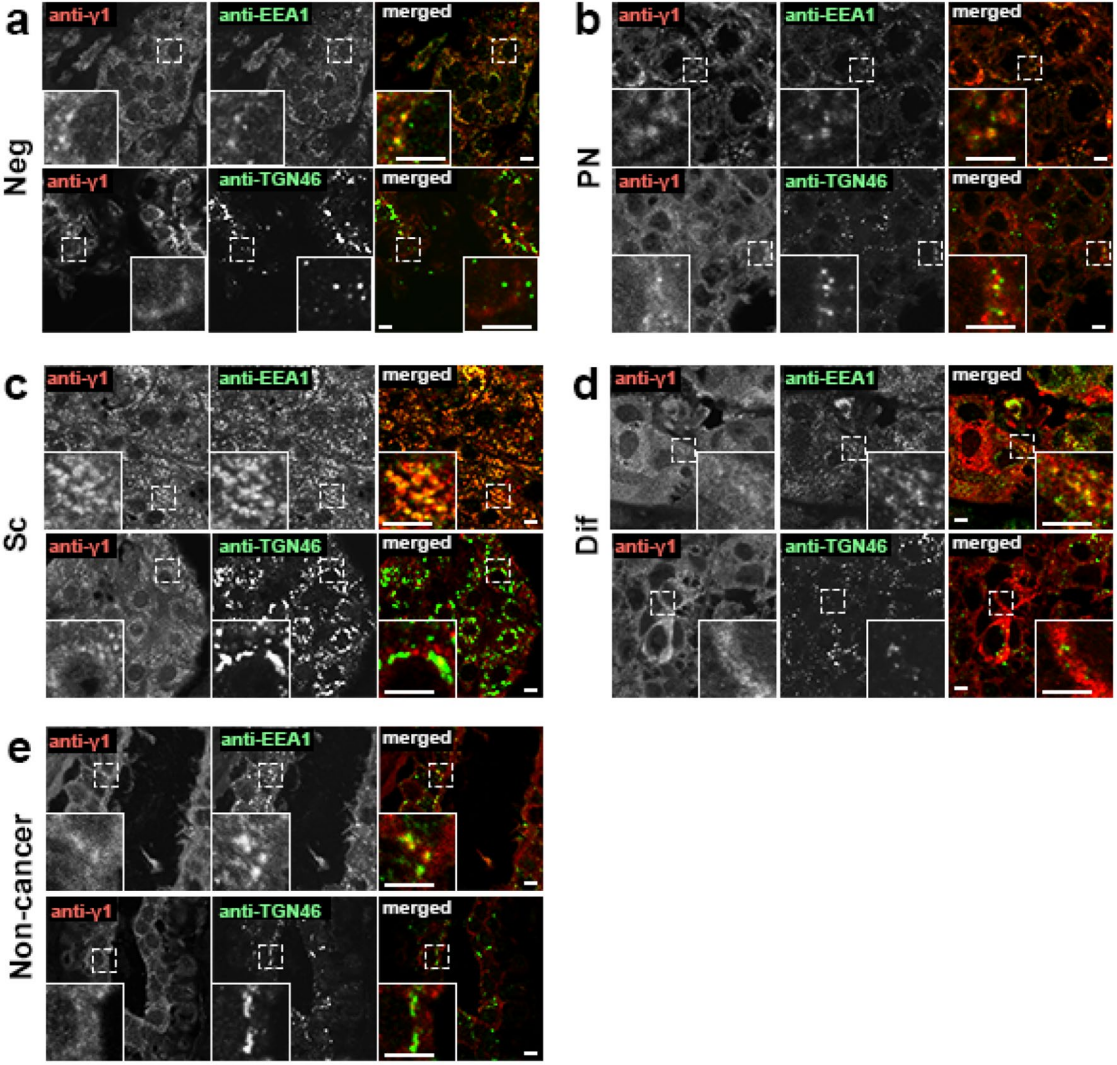
Subcellular localization of AP-1 in breast cancer tissues. Double IHF was performed in cases of Neg (**a**), PN (b), SC (**c**), and Dif (**d**), and non-cancer regions (**e**) using a combination of anti-γ1-adaptin (anti-γ1, red) and anti-EEA1 (endosomal marker, green), or anti-TGN46 (TGN marker, green). Merged images are shown on the right. Boxed regions are magnified and shown in insets. Bars: 5 µm

Since intracellular distribution of γ1-adaptin varied among breast cancer patients, we examined its detailed localization by double IHF microscopy using an endosomal marker, EEA1, and a TGN marker, TGN 46. Since this analysis allowed us to detect cytoplasmic puncta with higher sensitivity and resolution than conventional IHC, we could detect γ1-adaptin-positive puncta more clearly in all types of tissues. As shown in Fig. 3a, they often colocalized with EEA1, but only rarely with TGN46. Interestingly, the dominant endosomal localization of γ1-adaptin was observed even in the PN-type cancer cells (Fig. 3b) and ductal epithelial cells in non-cancer regions (Fig. 3e). In Dif-type cases, γ1-adaptin was still diffusely distributed in some cells (Fig. 3d), probably reflecting high cytoplasmic expression of γ1-adaptin and/or some artifacts from tissue processing prior to fixation. These observations indicate that the distribution patterns of γ1-adaptin do not reflect different organellar localizations of γ1-adaptin, but the different distribution pattern of γ1-adaptin-positive endosomes.

### Relationships between γ1-adaptin intensity and clinicopathological factors

**Fig. 4:**
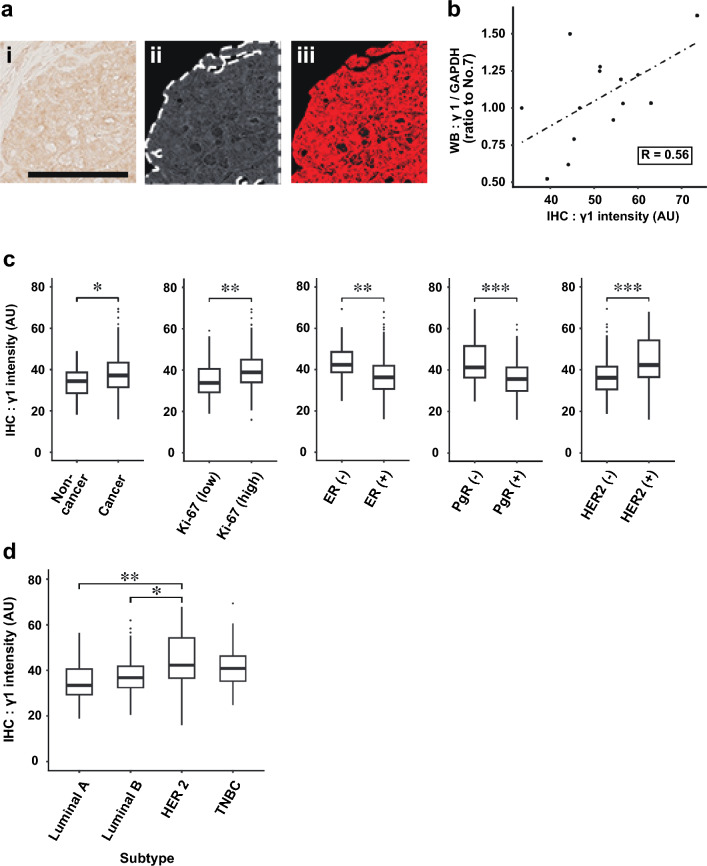
Quantification of γ1-adaptin intensity and comparison with clinicopathological factors. **a** In an original image of ROI (i), the area mainly containing tumor cells were manually enclosed (dotted line), converted to gray scale and color-inverted (ii). Extracted thresholded pixels are red (iii). **b** Correlation analysis between the γ1 adaptin intensities by IHC and values obtained from the Western blot analysis (WB) in Fig. 1b. **c** γ1-Adaptin intensity was compared between the cancer and non-cancer regions, and between groups divided according to Ki-67, ER, PgR, and HER2. *p < 0.05, **p < 0.01, ***p < 0.001 (Mann–Whitney *U* test). (d) γ1-adaptin intensity was compared among the subtypes. *p < 0.05, **p < 0.01 (Steel–Dwass test)

To confirm whether the staining intensity in IHC correlates with band intensity in Western blot analysis, we quantified both intensities in 14 breast cancer tissues. For quantification of IHC images, the intensity of the areas that exceeded a certain threshold value was measured (Fig. 4a). As expected, the IHC values were moderately correlated with those of the Western blotting (*r* = 0.56, Fig. 4b), indicating that the IHC values may be applicable for following analyses. The values in the cancer regions (199 cases) were significantly higher than those in the non-cancer regions (44 cases; Fig. 4c). Furthermore, they were significantly higher in patients with Ki-67 (high), ER (–), PgR (–), and HER2 (+) than in those with Ki-67 (low), ER (+), PgR (+), and HER2 (–), respectively (Fig. 4c). Among the subtypes, γ1-adaptin intensity was higher in HER2 than in luminal A or luminal B (Fig. 4d).

**Fig. 5:**
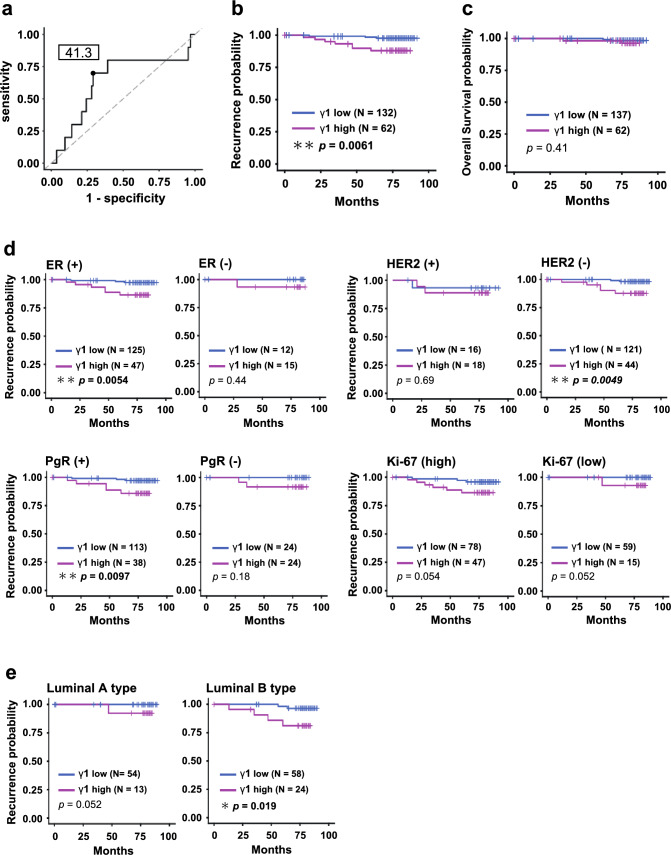
Survival time analysis. **a** ROC analysis for obtaining an optimum cut-off value for γ1-adaptin intensity. **b**, **c** Cases were divided into two groups by the cut-off value, and recurrent free survival (RFS; **b**) and overall survival (**c**) were evaluated using the Kaplan–Meier method. **d** Cases were divided into two groups according to ER, PgR, HER2, and Ki-67, and then, RFS was evaluated using the Kaplan–Meier method. **e** RFS was also analyzed for cases of luminal A and luminal B types. *p < 0.05, **p < 0.01 (log-rank test)

Next, a receiver-operating characteristic analysis was performed according to the presence or absence of metastasis or recurrence (Fig. 5a), and a cut-off value of 41.3 was determined for further analyses. The γ1-adaptin intensity was significantly associated with Ki-67, ER, PgR, HER2, and Subtype, but not with age, tumor size, axillary lymph-node metastasis, or stage (Table 1), supporting the results of Fig. 4c, d. Kaplan–Meier curves with log-rank test showed that RFS, but not overall survival, in patients with high γ1-adaptin intensity was significantly shorter than that in patients with low γ1-adaptin intensity (Fig. 5b). The poor RFS in high γ1-adaptin intensity was also observed in patients with ER (+), PgR (+), HER2 (–), and luminal B type (Fig. 5d, e). We also applied multivariate analysis of the Cox proportional hazards model to evaluate associations of RFS with γ1-adaptin intensity, tumor size, lymph-node metastasis, Stage, ER, PgR, HER2, and Ki-67 (Table 2). γ1-Adaptin alone was significantly associated with RFS, indicating that γ1-adaptin can be considered an independent prognostic factor.

**Table 1:** Correlation between γ1 intensity and clinicopathological factors

Factors	γ1 intensity^a^		
	Low (*N* = 137)	High (*N* = 62)	*p* value^b^
Age			0.282
< 60	79	30	
≥ 60	58	32	
Tumor size (cm)			0.874
≤ 2	88	39	
> 2	49	23	
Lymph-node metastasis			0.866
Positive	40	17	
Negative	97	45	
Stage			0.761
1	72	31	
2 + 3	65	31	
ER			**0.006
Positive	125	47	
Negative	12	15	
PgR			**0.002
Positive	113	38	
Negative	24	24	
HER2			**0.004
Positive	16	18	
Negative	121	44	
Ki-67			*0.012
< 14	59	15	
≥ 14	78	47	
Subtype			**0.004
Luminal A	54	13	
Luminal B	58	24	
HER2	16	18	
TNBC	9	7	

*p < 0.05, **p < 0.01

^a^cut-off value = 41.3

^b^Chi-square test

**Table 2:** Hazard ratios of prognostic variables with Cox proportional hazard model

Variables	Hazard ratio (95% CI)	Unfavorable/Favorable	*p* value
Univariate analysis			
γ1 intensity	5.41 (1.39–20.93)	High/low^a^	*0.014
Tumor size	7.16 (1.52–33.73)	> 2 cm/ ≤ 2 cm	*0.012
Lymph-node metastasis	1.54 (0.43–5.46)	Positive/negative	0.50
ER	1.26 (0.15–9.96)	Positive/negative	0.82
PgR	1.21 (0.25–5.72)	Positive/negative	0.80
HER2	2.12 (0.55–8.23)	Positive/negative	0.27
Ki-67	5.38 (0.68–42.48)	High/low^b^	0.11
Stage	4.19 (0.89–19.75)	2 + 3/1	0.069
Multivariate analysis			
γ1 intensity	5.50 (1.38–21.9)	High/low^b^	*0.016
Tumor size	5.49 (1.11–27.0)	> 2 cm/ ≤ 2 cm	0.12
Lymph nodes metastasis	1.11 (0.248–5.0)	Positive/negative	0.89
ER	1.10 (0.08–14.7)	Positive/negative	0.93
PgR	2.35 (0.29–19.1)	Positive/negative	0.43
HER2	1.24 (0.27–5.68)	Positive/negative	0.78
Ki-67	3.08 (0.35–26.4)	High/low^b^	0.31
Stage	0.73 (0.057–9.2)	2 + 3/1	0.81

*p < 0.05

^a^cut-off value = 41.3

^b^cut-off value = 14

## Discussion

In the present study, we evaluated the protein expression of γ1-adaptin in breast cancer tissues by IHC, and found that γ1-adaptin expression is higher in breast cancer regions than in non-cancer regions, and that higher expression of γ1-adaptin is well associated with Ki-67, ER/PgR-negativity, HER2-positivity, and poor RFS. Furthermore, the Cox proportional hazards model indicated that γ1-adaptin expression could be an independent prognostic factor for breast cancer. Previous analysis using the Cancer Genome Atlas (TCGA) demonstrated that overexpression of *AP1S3* is associated with poor outcomes in breast cancer, and the staining pattern of the *AP1S3* products is similar to the scattered pattern of γ1-adaptin in this study [16]. Thus, overall expression of AP-1 in endosomes is likely to be relevant to the prognosis in breast cancer. It may also be noted from our prognostic analysis that patients with high γ1-adaptin expression showed poor outcome even in ER (+), PgR (+), HER2 (–), and luminal B type (Fig. 5d, e). Testing for γ1-adaptin expression may therefore influence treatment decision in those patients.

It has been shown that high expression of a subunit of AP-1, µ1A-adaptin, mediated by transfection of hepatitis B virus promotes cell proliferation in HepG2 cells [14], and that AP-1 (µ-adaptin) is required for the growth of plant cells in Arabidopsis [23]. Moreover, depletion of one of AP-1 subunit was found to inhibit proliferation of several cancer-derived cell lines, including glioma cell lines, SW1783 and U373 [18], a lung cancer cell line, H1975 [13], and a TNBC cell line, MDA-MB-231 [16]. Notably, a recent study of searching microRNA in TNBC identified *AP1S3* as one of targets of *miR-204-5p*, and showed that low expression of *miR-204-5p* in MDA-MB-231 cells causes high expression of *AP1S3* that encodes σ1C-adaptin, supporting proliferation, migration, and invasion of the cells [16]. This and similar reports further demonstrated that the *AP1S3* transcript is highly expressed in TNBC, PDAC and glioma [16–18]. We also demonstrated in this study that depletion of γ1-adaptin in HER2-positive SK-BR-3 cells suppressed cell proliferation, migration, and invasion, and that there was a significant association between γ1-adaptin expression and the proliferation marker Ki-67 (Fig. 4c and Table 1). Therefore, it is most likely that higher AP-1 expression supports cancer cell activities.

However, previous studies did not show how AP-1 can promote cell proliferation. Regarding this, the present study demonstrated the preferential endosomal localization of γ1-adaptin in all types of distribution pattern (Fig. 3). We have previously found that endosomal localization of γ1-adaptin was remarkable also in the NSCLC, hepatocellular carcinoma, and colorectal cancer tissues [13]. Although accumulating evidence indicates that AP-1 localizes in both the TGN and endosomes in culture cells [10, 11], the predominant localization of γ1-adaptin in endosomes observed in the present study may be characteristic in human tissues. It should be noted that the different distribution patterns of AP-1 do not necessarily reflect different localizations of AP-1, e.g., in the TGN or endosomes, but may reflect the distribution of endosomes. We therefore propose that endosome-localizing AP-1 could be relevant for cancer cell malignancy.

Previous reports have suggested that AP-1 functions in endosomal sorting of important cell surface receptors, such as transferrin receptor, low-density lipoprotein receptor, and EGFR [10–13]. Moreover, our recent study showed that the cell surface fractions of Erb-B2 (HER2), MET, and IR, as well as EGFR are decreased by AP-1 knockdown in culture cells, which can be explained by the reduced rate of EGFR recycling from endosomes back to the cell membrane, which causes an accelerated degradation of EGFR in lysosomes [13]. Thus, it was proposed that AP-1 plays a key role in maintaining cell surface receptors such as EGFR [13]. Interestingly, it is known that EGFR can form a heterodimer with HER2 [24], regulating their diverse signaling network. Therefore, it is possible that AP-1 regulates HER2 expression via EGFR. Indeed, EGFR-positive tumors co-expressed HER2 to varying degrees [25–28]. We infer that this could explain the higher expression of γ1-adaptin in HER2 (+) patients (Fig. 4c). It has also been reported that TNBC with brain metastasis expresses high levels of µ1-adaptin (AP1M1) [15]. This may reflect a high rate of EGFR positivity in the TNBC group [29–32], though involvement of other RTKs cannot be excluded.

In the present study, however, we could not elucidate detailed molecular mechanisms of how AP-1 acts in endosomes. EGFR recruits AP-1 via its cytoplasmic domain [12, 13]; thus, investigation into the relationship between endosomal AP-1 and EGFR in breast cancer cells is required in future. Furthermore, exploring additional binding partners of AP-1 will advance our understanding of the function of AP-1 at endosomes.

## Data Availability

The data that support the findings of this study are available from the corresponding author upon reasonable request.
